# Impaired Modulation of Motor and Functional Performance in Patients after Total Knee Arthroplasty: A Prospective Observational Study

**DOI:** 10.1155/2022/4546836

**Published:** 2022-08-28

**Authors:** Federico Temporiti, Davide De Leo, Paola Adamo, Gabriele Papa, Francesco Traverso, Nicola Maffiuletti, Roberto Gatti

**Affiliations:** ^1^Department of Biomedical Sciences, Humanitas University, 20072 Pieve Emanuele, Milan, Italy; ^2^Physiotherapy Unit, IRCCS Humanitas Research Hospital, 20089 Rozzano, Milan, Italy; ^3^Hip Diseases and Joint Replacement Surgery Unit, IRCCS Humanitas Research Hospital, 20089 Rozzano, Milan, Italy; ^4^Human Performance Lab, Schulthess Clinic, 8008 Zurich, Switzerland

## Abstract

Submaximal levels of effort are required for the performance of the most common daily tasks. Inaccuracy in modulating motor outputs during submaximal tasks has been reported as indicator of safety during daily activities in subjects with lower limb musculoskeletal disorders. The study is aimed at investigating performance modulation ability during motor and functional tasks in patients after total knee arthroplasty (TKA). Sixteen patients with end-stage osteoarthritis undergoing TKA and twenty age-matched healthy participants performed isokinetic knee extension, sit-to-stand, and walking tasks at three levels of self-estimated effort (100%, 50%, and 25%) the day before (T0) and 4 days after surgery (T1). Maximum performance in terms of peak torque (PT—knee extension), overshoot (OS—sit-to-stand), and walking speed was evaluated. Subsequently, relative error (RE) between target and observed performance was computed for the submaximal tasks (RE_50%_ and RE_25%_). Our results showed a decline of maximum performance after surgery, which resulted lower in patients compared to healthy subjects. RE_50%_ and RE_25%_ for knee extension (involved limb) (*p* < 0.001) and RE_25%_ for sit-to-stand (*p* < 0.001) increased from pre- to postsurgery. At T0, knee extension RE_25%_ and walking RE_50%_ and RE_25%_ were higher in patients. At T1, RE_50%_ and RE_25%_ were higher in patients for knee extension (involved limb), sit-to-stand, and walking. In conclusion, the ability to modulate motor and functional performance decreased after TKA and resulted impaired when compared to healthy age-matched subjects. Based on relationship between ability to modulate motor outputs and risk of falling, the role of modulation ability as indicator of readiness for discharge and safe return to daily activities deserves further investigations in patients in early phase after TKA.

## 1. Background

Total knee arthroplasty (TKA) represents an effective and definitive treatment for end-stage knee osteoarthritis, able to relieve pain, improve functional level, and quality of life [1]. The main goal of rehabilitation after TKA is to rapidly achieve functional independence to reduce length of stay and allow for an early return to daily activities [2]. However, a successful execution of daily tasks also requires an adequate ability to modulate motor performance, which represents a measure of neuromuscular control [3, 4].

Modulation of motor output has been reported to depend on central and peripheral processes in relation to sense of force or tension and sense of effort [5]. Sense of force originates from articular, muscular, and cutaneous receptors, whereas sense of effort arises from the perception of descending motor commands [6–8]. However, the contribution of these two mechanisms on performance modulation ability remains controversial, especially in patients with musculoskeletal impairments (e.g., end-stage knee osteoarthritis) or after surgery (e.g., total knee arthroplasty), for whom muscle and/or joint damages generate a discrepancy between sense of force and sense of effort [5, 9, 10].

Performance modulation ability can be estimated by computing the error between target and generated performance during motor tasks executed at preestablished submaximal levels [11–13]. Studies reported impairments in developing specific submaximal force levels in older adults and patients with musculoskeletal disorders [14]. A single study demonstrated impaired ability of patients with knee osteoarthritis to accurately exert submaximal force levels during concentric and eccentric isokinetic knee extension tasks [15]. However, no studies assessed ability to modulate motor performance during functional tasks in patients with end-stage knee osteoarthritis undergoing TKA. In fact, studies investigating motor and functional recovery after TKA mainly focused on restoration of maximal motor performance, which can be influenced by perceived pain or motivation and seems not to be a strong predictor of difficulties experienced by these patients during daily activities [16–20]. Indeed, the more common daily tasks usually require submaximal effort levels, and inaccuracy in modulating motor outputs, such as force, during lower limb tasks has been reported as related to increased risk of falling [14, 21]. In fact, the ability to accurately modulate motor performance is essential to enable adaptations to continuous environmental changes typical of daily activities [14]. Consequently, when considering patients in early phase after TKA, it is reasonable to speculate that, in addition to maximal performance, modulation ability during motor and functional tasks might be indicative of readiness for discharge and safe return to daily activities. Therefore, the aim of this study was to evaluate the ability to modulate performance during motor and functional tasks in patients after TKA.

## 2. Methods

### 2.1. Participants

Twenty patients scheduled for unilateral TKA and twenty healthy age-matched volunteers were enrolled in this study between March and October 2019. Patients satisfied the following inclusion criteria: age between 40 and 80 years, TKA due to primary knee osteoarthritis, passive knee flexion ≥90° and complete knee extension, ability to stand up from a standard chair without the use of upper limbs, and ability to walk for at least 50 meters without aids. Exclusion criteria were previous TKA on contralateral limb or lower extremity disorders that would have required surgery (e.g., anterior cruciate ligament reconstruction, meniscal repair, and total hip arthroplasty), previous tibial and/or femoral osteotomy, revision surgery and concomitant neurological and/or musculoskeletal disorders influencing motor, and functional recovery. Healthy participants were enrolled from among employees of our Institute or patients' relatives and had to satisfy all the aforementioned eligibility criteria except “TKA for primary osteoarthritis.” Patients were operated by the same orthopaedic surgeon using the same surgical technique (mid-vastus approach). The prosthetic components (Zimmer Biomet, USA) were aligned using the mechanical alignment technique, and the gap between extension and flexion and soft tissues balance was ensured through the original gap technique in all participants. After surgery, patients followed a standardized in-hospital rehabilitation program consisting of two daily sessions focused on recovery of functional independence and daily-living activities such as transfers, walking with crutches, and stair climbing. Rehabilitation also included progressive resistance training and exercises aimed at improving knee range of motion (ROM), endurance, and postural stability. The study was conducted at the Hip and Knee Orthopaedic Surgery Department and the Motion Analysis Laboratory of Humanitas Research Hospital. The procedures followed were in accordance with ethical standards of the ethical committee on human experimentation that approved the study protocol (Humanitas Clinical and Research Center Committee, approval number CLF 18/03) and with Helsinki Declaration. All participants signed a written informed consent form, and the study was registered at ClinicalTrials.gov (protocol registration number NCT03997565, 25/06/2019).

### 2.2. Study Design

This was a prospective observational study. Patients were evaluated the day before (T0) and 4 days after surgery (T1), whereas healthy participants underwent a single evaluation session. All participants were asked to perform three different tasks in the following sequence: isokinetic knee extension, sit-to-stand (STS), and walking (10-meter walking test -10MWT). Two series of three repetitions (maximum performance, 50% and 25% of the maximum performance performed according to participants' perception) were performed for knee extension and STS tasks, whereas only one set of three repetitions was executed for the 10MWT to reduce the risk of fall. The submaximal levels were adopted in order to explore the modulation ability in a range of intensity equal to or lower than 50% of the maximum performance, which has been documented to characterize the more common daily tasks [21, 22]. The three tasks were separated by 5 minutes of rest and, before each task, participants had to indicate a perceived exertion lower than 2 points on the Modified Rate of Perceived Exertion scale (range 0-10) in order to minimize the impact of fatigue on performance. In addition, a rest period of 15 seconds interspaced the repetitions within each task. The same physiotherapist performed all the assessments using highly-standardized procedures and instructions. For all the tasks, no feedback was provided to participants about their performance.

Each session started with the knee extension task (90°-0° ROM) that was performed using an isokinetic dynamometer (Prima Doc, Easytech, Italy) at an angular velocity of 60°/s. The uninvolved limb was always tested before the involved one. Participants were seated upright on the dynamometer chair, with hips and knees at 90° and secured using with seatbelts. The dynamometer rotational axis was aligned with the lateral femoral condyle, and the lever arm was fixed about 3 cm above the lateral malleolus using an inextensible band with Velcro straps. Participants were asked to extend the knee with as much strength as possible and, subsequently, to reproduce the task at a self-estimated level of 50% and 25% of the maximum force. The STS task was performed using a chair without armrests that was placed adjacent to a force platform (BTS P-6000, BTS, Italy). Participants were seated upright on the chair, with hips and knees at 90° of flexion (verified with a manual goniometer) and feet equidistant (intermalleolar distance of 20 cm) over the force plate. They were asked to stand up from the chair as quickly as possible and, subsequently, to reproduce the task at a self-estimated level of 50% and 25% of the maximum speed. Finally, for the 10MWT, participants were asked to walk for 10 meters as quickly as possible without aids, and subsequently, reproduce the task at a self-estimated level of 50% and 25% of the maximum speed. The performance was timed with a stopwatch always by the same physiotherapist.

### 2.3. Measures of Performance Modulation

**Figure 1:**
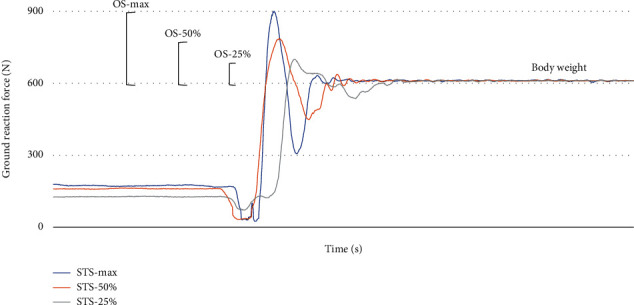
Overshoot during sit-to-stand task. Vertical ground reaction force (GRF) of a representative subject during sit-to-stand performed at maximum speed and 50% and 25% of the maximum speed. Overshoot (OS) was calculated for each repetition (maximum, 50% and 25% of the maximum) as the difference between GRF-peak and body weight.

The maximum performance was expressed in terms of peak torque (PT) with the relative angle of occurrence for the knee extension task, overshoot (OS_max_), described as the difference between the peak of vertical ground reaction force and body weight for the STS task (Figure 1) and speed (Speed_max_) for the walking task. At the end of each maximal task, the self-reported level of knee pain was assessed using a 0-10 visual analogue scale (VAS).

The ability to modulate performance was estimated through the relative error (RE) (equation (1)) at 50% (RE_50%_) and 25% (RE_25%_) of the maximum performance, expressed as [11]

(1) $$\begin{array}{c} \text{RE}=\left|\frac{\text{target performance}-\text{observed performance }}{\text{target performance }}\right|\;\times \;100, \end{array}$$

where the target performance is the force, OS, and speed values corresponding to the requested percentage of the maximum, whereas the observed performance represents the force, OS, and speed generated by participants. Lower RE values indicate higher modulation ability [11].

For the knee extension task, RE_50%_ and RE_25%_ were calculated using the torque data recorded at the same angle as PT. For the STS and walking tasks, RE was calculated using submaximal OS (Figure 1) and speed data, respectively. When considering STS, OS was adopted as performance index based on its relationship with chair rise time and physical performance [23, 24]. Moreover, since speed varies among STS phases, changes in body accelerations exerted on the ground during the rising phase (e.g., OS) can be considered as indicative of variations in STS performance [24]. Finally, all participants filled out the Knee Injury and Osteoarthritis Outcome Score (KOOS) and the International Physical Activity Questionnaire (IPAQ) once (at T0 for patients) to estimate, respectively, the impact of knee symptoms on activities of daily living and physical activity level.

### 2.4. Statistical Analysis

Sample size was estimated based on RE during knee extension task with involved limb. It was estimated that, considering a two-tailed alpha error of 5% and allowing for a 20% of attrition rate, a minimum of 20 participants with TKA were required to provide 80% power to detect a large effect size (Cohen's *d* = 0.8) from pre- to postsurgery [25].

Categorical variables were described as proportions, whereas continuous variables were described as means and standard deviations. Two-tailed unpaired *t*-tests or Chi-square tests were used to investigate differences between patients and healthy participants in terms of baseline characteristics. Mixed-model ANOVAs with “time” as within-subject factor and “limb” as between-subject factor were used to assess differences between the involved and uninvolved limb from T0 to T1 in patients for PT, RE_50%_, and RE_25%_ during the knee extension task. Significant interactions were analysed step by step using simple effect analyses, whereas post hoc analyses of significant main effects were performed using two-tailed *t*-tests with Bonferroni correction for multiple comparisons. Additionally, two-tailed paired *t*-tests were used to assess differences from T0 to T1 in patients for OS_max_, Speed_max_, VAS at involved-limb PT, VAS at uninvolved-limb PT, VAS at OS_max_, VAS at Speed_max_, and RE_50%_ and RE_25%_ for STS and 10MWT. Effect size from T0 to T1 in patients was also quantified for all outcome measures using Cohen's *d* and considered small (0.2), medium (0.5), or large (≥0.8) [25]. Moreover, two-tailed unpaired *t*-tests with Bonferroni correction for multiple comparisons were used to compare all outcome measures between healthy participants and patients at both T0 and T1.

Finally, Pearson's correlation coefficients were used to assess any correlation between the maximum value, RE_50%_, and RE_25%_ of each task and patients' clinical outcome measures (VAS, IPAQ and KOOS). The strength of correlation was interpreted as small, moderate, and strong for <0.3, 0.3-0.6, and >0.6, respectively [26]. Statistical analyses were performed using SPSS 25.0 software for Windows, and the statistical level of significance was set at *α* = 0.05.

## 3. Results

All participants completed the evaluation correctly at T0, whereas four patients were excluded from the study due to the inability to extend the knee completely at T1. Patients and healthy participants were homogeneous in terms of age, gender, weight, height, dominant limb, and IPAQ score, whereas a between-group difference was found for KOOS score (Table 1).

### 3.1. Maximal Performance

A time by limb interaction (*p* < 0.001) as well as a main effect of limb (*p* = 0.003) and time (*p* < 0.001) was found for PT. Simple effect analysis for interactions revealed that involved-limb PT significantly decreased from T0 to T1 (*p* < 0.001, *d* = 1.7) (Table 2), unlike uninvolved-limb PT, which did not change from pre- to postsurgery (*p* = 0.631) and resulted significantly higher than involved-limb PT at T1 (*p* < 0.001). Furthermore, OS_max_ (*d* = 1.3) and Speed_max_ (*d* = 1.7) declined significantly, whereas VAS-OS_max_ increased from pre- to postsurgery in patients (Table 2). Finally, involved-limb PT, uninvolved-limb PT, OS_max_, and Speed_max_ scores were significantly lower both before and after surgery in patients than in healthy participants (Table 2).

### 3.2. Performance Modulation: Pre- versus Postsurgery

**Figure 2:**
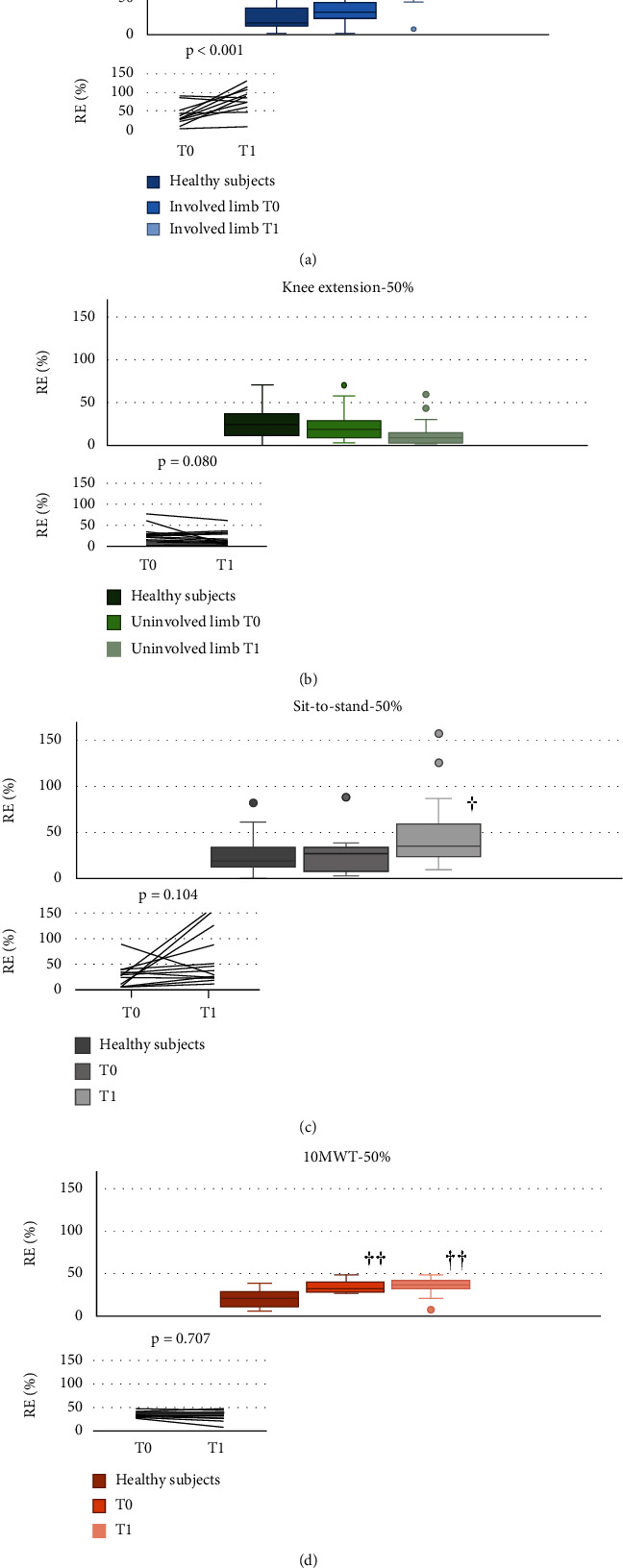
RME50% for the different tasks by group, time, and side. (a) Knee extension involved side. (b) Knee extension uninvolved side. (c) Sit to stand. (d) 10MWT. The box represents the range between the first and the third quartile, the horizontal line represents the median value, and the two whisker boundaries represent the maximum and minimum values (outliers are also shown). The inset shows individual changes from T0 to T1 in patients. ^†^*p* < 0.05, ^††^compared to healthy subjects.

**Figure 3:**
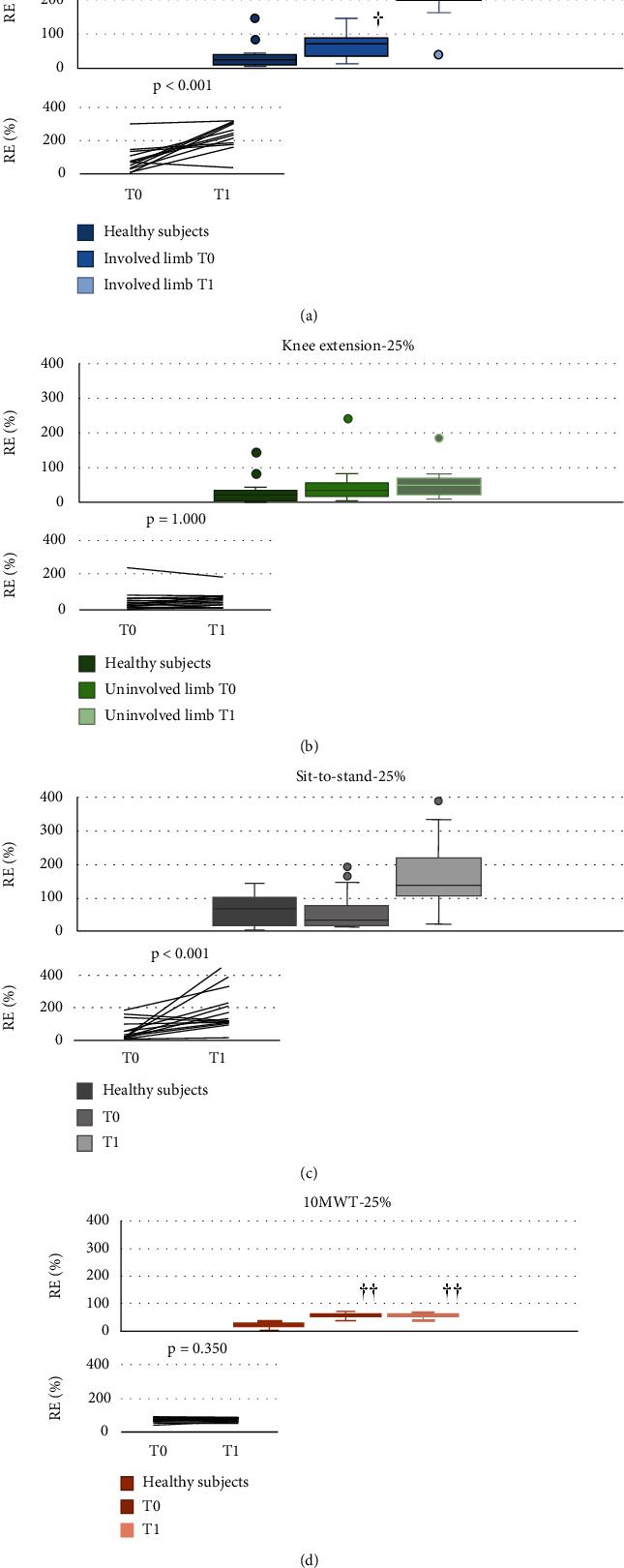
RME25% for the different tasks by group, time, and side. (a) Knee extension involved side. (b) Knee extension uninvolved side. (c) Sit to stand. (d) 10MWT. The box represents the range between the first and the third quartile, the horizontal line represents the median value, and the two whisker boundaries represent the maximum and minimum values (outliers are also shown). The inset shows individual changes from T0 to T1 in patients. ^†^*p* < 0.05, ^††^compared to healthy subjects.

A time by limb interaction (*p* < 0.001) as well as a main effect of limb (*p* < 0.001) and time (*p* < 0.001) was found for RE_50%_ and RE_25%_ during knee extension. Simple effect analysis for interactions revealed that RE_50%_ and RE_25%_ during knee extension with involved-limb significantly increased from T0 to T1 (*d* = 1.6 for RE_50%_ and *d* = 2.1 for RE_25%_) (Figures 2(a) and 3(a)), unlike RE_50%_ and RE_25%_ during knee extension with uninvolved-limb, which did not change from pre- to postsurgery (Figures 2(b) and 3(b)). RE_25%_ increased significantly from T0 to T1 during STS (*d* = 0.9) (Figure 3(c)), whereas no significant differences were found for RE_50%_ during STS and RE_50%_ and RE_25%_ during walking task (Figures 2(c), 2(d), and 3(d)).

### 3.3. Performance Modulation: Patients versus Healthy Participants

Patients showed significantly higher involved-limb knee extension RE_50%_ at T1 and RE_25%_ at T0 and T1 compared to healthy participants (Figures 2(a) and 3(a)), whereas no between-group differences were observed for the uninvolved limb both before and after surgery (Figures 2(b) and 3(b)). Moreover, both RE_50%_ and RE_25%_ during STS were significantly higher at T1, but not at T0, in patients compared to healthy participants (Figures 2(c) and 3(c)). Finally, patients showed higher walking RE_50%_ and RE_25%_ than healthy participants both before and after surgery (Figures 2(d) and 3(d)).

### 3.4. Correlations with Clinical Outcome Measures

Significant correlations were observed between KOOS and PT (*r* = 0.634, *p* = 0.020), RE_50%_ (*r* = −0.697, *p* = 0.008), and RE_25%_ (*r* = −0.653, *p* = 0.016) for the knee extension task with the involved limb at T0, but not at T1. No correlations were found for maximum value, RE_50%_, and RE_25%_ related to each task with IPAQ and VAS both before and after surgery.

## 4. Discussion

The main findings of this study were that performance modulation ability during knee extension with involved limb and STS tasks decreased after TKA and were lower with respect to healthy age-matched participants. Moreover, modulation ability of walking was lower in patients, when compared to healthy participants both before and after surgery.

Deficits in performance modulation ability such as those observed in this study for knee extension and sit-to-stand tasks might derive from alterations of ascending sensory information and perception of feedforward neural signals responsible for sense of force and sense of effort [3, 9, 10]. When considering force modulation ability, sense of force originates from peripheral receptors, which undergo anatomical damage with joint degeneration and further deteriorate after joint replacement surgery [7]. Previous studies demonstrated a role of proprioceptive deficits on impaired modulation of motor outputs in older adults and in patients with complex regional pain syndrome, which resulted in higher levels of performance than in those required during force-control tasks [11, 13]. Older adults revealed REs of approximately 150% during the exertion of forces equal to 20% of the maximal voluntary contraction with dorsal and plantar flexor ankle muscles, whereas patients with complex regional pain syndrome (disease duration > 10 years) showed RE values ranging from 150% to 50% during hand-grip force modulation tasks at 1, 3, and 5 N [11, 13].

Our results are in contrast with the findings of Carson et al., who reported preserved force modulation ability for a force-matching task after experimentally-induced muscle damage, suggesting an exclusive reliance on sense of effort in the presence of impaired sensory feedback [5]. However, the strategy that induces individuals with sensation deficits to rely on the perception of descending motor commands may not apply to our patients, whose efferent signals were probably impaired by activation failure, as suggested by the considerable reduction of PT in the first days after TKA [27]. The abnormal efferent discharge occurring in patients with activation failure after knee surgery is characterized by a disruption of afferent excitatory signals to *γ*-motoneurons, which may attenuate the facilitatory discharge to the *α*-motoneuron pool (*γ*-loop dysfunction). As documented by Luu et al., decoupling the *α* − *γ* linkage may generate a bias in sense of effort, leading these patients to develop higher levels of performance than those required during submaximal tasks with the involved limb [18]. As proposed by several authors, sense of force and sense of effort do not seem to be two stand-alone underlying mechanisms and a role of sensory input from peripheral structures in modulating sense of effort, especially in patients with articular degenerative conditions or after joint replacement surgery, cannot be excluded [28].

Interestingly, performance modulation ability has also been suggested to depend on the amount of maximum performance. In fact, modulation of predefined levels of force has been found to be more accurate for participants with higher maximum voluntary strength when compared to its lower equivalent [6, 29, 30]. In particular, patients with knee osteoarthritis showed RE values of approximately 30% and 10% during a concentric knee extension performed at 50 N and 100 N, respectively, demonstrating that accuracy in force generation increases with increasing force levels [15]. Coherently, in our study, modulation ability was more accurate in the presence of higher levels of performance (i.e., in healthy participants and in patients before surgery). Moreover, the correlation between knee extension RE_50%_ and RE_25%_ and KOOS at baseline also suggested a relationship between force modulation capacity and knee function during daily activities.

Patients revealed decreased performance modulation ability also during the STS task, where impairments in modulating performance of the involved limb might have influenced the correct execution of this functional task. Tsuji et al. suggested an association between ground reaction force parameters (e.g., peak force normalized to body weight) and lower limb muscle strength during STS, leading to speculate that this may also apply to submaximal efforts [31]. However, in our patients, the ability to modulate STS performance decreased after surgery, although both lower limbs were involved in the task. In fact, although development of adaptive strategies with the uninvolved limb have been described in patients with unilateral joint replacement in the first week after surgery, the healthy side does not seem to be enough to perform the requested task as in healthy participants [32]. In particular, the execution of this functional task seems to be mainly controlled by the involved limb. Pain increased after surgery during the STS, even if the observed levels can be considered too low to influence motor performance [33].

When considering walking, patients revealed an impaired ability to modulate speed with respect to healthy participants. The ability to vary speed during walking has been described as important for ensuring postural stability during daily tasks, where continuous gait speed adjustments in relation to environmental changes are required [34]. Coherently, in our study, walking speed modulation ability was reduced in patients before and after TKA, in which balance deficits and increased risk of falling have been described, especially in the first days after surgery [35].

Some limitations of this study need to be underlined. Participants' fear of movement was not assessed but might have influenced the execution of the different tasks. We speculated that activation failure might have influenced sense of effort, but this variable was not objectively quantified. Furthermore, no significant differences were found in IPAQ score between patients and healthy participants. However, whereas patients' IPAQ score was similar to those reported in literature for the considered population, healthy participants reported lower physical activity levels, when compared to normative data [36, 37]. Finally, the short-term follow-up did not allow us to monitor the modulation ability recovery after TKA.

## 5. Conclusions

Patients revealed decreased performance modulation ability after TKA surgery, which was also lower with respect to healthy participants both before and after surgery. The execution of daily-living activities requires submaximal levels of performance. Therefore, based on the relationship between ability to control motor outputs during lower limb submaximal tasks and risk of falling, performance modulation of motor and functional tasks might represent an indicator of readiness for discharge and safe return to daily activities just a few days after surgery [11]. In this scenario, future studies investigating this relationship would be informative for implementing rehabilitative plans in patients after TKA, where interventions addressed to recalibrate sense of force, sense of effort, and their interaction, such as symmetry-based resistance training or sensorimotor interventions incorporating biofeedback, might be proposed to improve performance modulation ability in early postoperative phase.

## Figures and Tables

**Table 1:** Characteristics of patients and healthy participants.

	HP (*n* = 20)	TKA (*n* = 20)	*p* value (TKA vs. HP)
Age (years)	62 ± 6	66 ± 8	0.157
Sex	11 M/9 W	8 M/12 W	0.342
Weight (kg)	73 ± 12	78 ± 17	0.267
Height (cm)	169.3 ± 6.4	166 ± 11	0.239
Dominant limb	20R/0 L	20R/0 L	1
KOOS	91 ± 10	47 ± 11	<0.001
IPAQ	2791 ± 1991	2669 ± 3652	0.905

Legend. Data are shown as mean ± standard deviation. *p* values refer to the comparison between groups (unpaired *t*-test or Chi-square test). Abbreviations. TKA: total knee arthroplasty; HP: healthy participants; M: men; W: women; R: right; L: left; KOOS: knee injury and osteoarthritis outcome; IPAQ: International Physical Activity Questionnaire.

**Table 2:** Maximal performance for respective tasks with the associated pain level by group and time-point.

	HP (*n* = 20)	TKA (*n* = 16)		*p* value (T0 vs. T1 in TKA)
	T0	T0	T1	
PT involved (nm) PT angle (°)	100 ± 27^∗∗^^§§^ (51 ± 8)	51 ± 22 (54 ± 21)	23 ± 5 (34 ± 15)	<0.001
PT uninvolved (nm) PT angle (°)	100 ± 27^∗∗^^§§^ (51 ± 8)	65 ± 30 (51 ± 14)	63 ± 28 (49 ± 13)	0.631
OS_max_ (*N*)	269 ± 90^∗^^§§^	190 ± 82	92 ± 71	<0.001
Speed_max_ (m/s)	2.1 ± 0.2^∗∗^^§§^	1.4 ± 0.4	0.8 ± 0.3	<0.001
VAS-PT involved (0-10)	0.0 ± 0.2^∗∗^^§§^	2.8 ± 2.3	4.2 ± 2.9	0.121
VAS-PT uninvolved (0-10)	0.0 ± 0.2^∗^^§^	1.0 ± 2.0	0.8 ± 1.4	0.667
VAS-OS_max_ (0-10)	0.1 ± 0.2^∗^^§§^	1.2 ± 1.9	2.6 ± 2.1	0.032
VAS-Speed_max_ (0-10)	0.7 ± 0.3^∗^^§^	1.8 ± 2.7	1.8 ± 2.1	1.000

Legend. Data are shown as mean ± standard deviation. *p* values refer to the comparison between T0 and T1 (simple effect analysis for ANOVA interactions for PT involved and PT uninvolved, paired *t*-test with Bonferroni correction for OS_max_, Speed_max_, and VAS during all tasks). Differences between groups are also shown (unpaired *t*-test with Bonferroni correction). Abbreviations. TKA: total knee arthroplasty; HP: healthy participants; PT: peak torque during knee extension; OS_max_: maximum overshoot during STS; Speed: maximum speed during 10MWT; VAS: visual analogue scale. Symbols legend: ^∗^HS vs. TKA at T0 (*p* < 0.05); ^∗∗^HS vs. TKA at T0 (*p* < 0.001); ^**§**^HS vs. TKA at T1 (*p* < 0.05); ^**§§**^HS vs. TKA at T1 (*p* < 0.001).

## Data Availability

The datasets used to support the findings of this study are available from the corresponding author upon request.
